# Symbiotic, low-temperature, and scalable synthesis of bi-magnetic complex oxide nanocomposites†

**DOI:** 10.1039/c9na00619b

**Published:** 2020-01-16

**Authors:** F. Sayed, G. Kotnana, G. Muscas, F. Locardi, A. Comite, G. Varvaro, D. Peddis, G. Barucca, R. Mathieu, T. Sarkar

**Affiliations:** Department of Materials Science and Engineering, Uppsala University Box 534 SE-75121 Uppsala Sweden tapati.sarkar@angstrom.uu.se; Department of Physics and Astronomy, Uppsala University Box 516 SE-75120 Uppsala Sweden; Dipartimento di Chimica e Chimica Industriale, Università degli Studi di Genova Via Dodecaneso 31 Genova 16146 Italy; Physics and Chemistry of Nanostructures (PCN), Ghent University Krijgslaan 281-S3 B9000 Gent Belgium; Istituto di Struttura della Materia – CNR Area della Ricerca di Roma1, Monterotondo Scalo RM 00015 Italy; Department SIMAU, University Politecnica delle Marche Via Brecce Bianche Ancona 60131 Italy

## Abstract

Functional oxide nanocomposites, where the individual components belong to the family of strongly correlated electron oxides, are an important class of materials, with potential applications in several areas such as spintronics and energy devices. For these materials to be technologically relevant, it is essential to design low-cost and scalable synthesis techniques. In this work, we report a low-temperature and scalable synthesis of prototypical bi-magnetic LaFeO_3_–CoFe_2_O_4_ nanocomposites using a unique sol-based synthesis route, where both the phases of the nanocomposite are formed during the same time. In this bottom-up approach, the heat of formation of one phase (CoFe_2_O_4_) allows the crystallization of the second phase (LaFeO_3_), and completely eliminates the need for conventional high-temperature annealing. A symbiotic effect is observed, as the second phase reduces grain growth of the first phase, thus yielding samples with lower particle sizes. Through thermogravimetric, structural, and morphological studies, we have confirmed the reaction mechanism. The magnetic properties of the bi-magnetic nanocomposites are studied, and reveal a distinct effect of the synthesis conditions on the coercivity of the particles. Our work presents a basic concept of significantly reducing the synthesis temperature of bi-phasic nanocomposites (and thus also the synthesis cost) by using one phase as nucleation sites for the second one, as well as using the heat of formation of one phase to crystallize the other.

## Abstract 2

Low-temperature, symbiotic synthesis of nanocomposites, with heat of formation of phase1 crystallizing phase2, and phase2 preventing grain growth of phase1.

## Graphical abstract

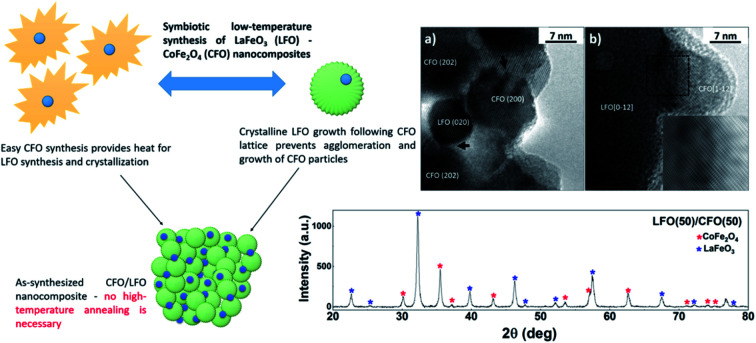

## 1. Introduction

Strongly correlated electron oxides form a very important class of materials where interactions between the electronic spins, charges, orbitals, and lattice co-exist simultaneously.^1^ This correlation typically produces a rich variety of states and leads to the occurrence of very many competing states in these materials.^2^ As a result, this family of oxides shows a plethora of properties such as colossal magnetoresistance,^3^ multiferroicity,^4^ high-*T*_c_ superconductivity,^5^ and spin-liquid states,^6^ to name a few. The existence of competing states with nearly equal energy scales of the dominant interactions also means that the ground state is extremely sensitive to doping and/or external perturbations such as electric and/magnetic field, pressure, temperature, and strain. The ground states of these oxides can be changed not only using external perturbations, but also by reducing the particle size to the nano regime. Nanoparticles, with larger surface to volume ratios and quantum confinement effects, possess distinct size-dependent optical, electronic, and magnetic properties. The effects of size reduction on the properties of strongly correlated electron oxides have been studied extensively over the last two decades, and are relatively well-established.^7–13^ More importantly, this family of oxides is interesting not only from the point of fundamental studies, but also due to potential applications in different areas of technology.^14,15^

In the quest for new systems with tunable properties, an alternative route that is being increasingly explored in recent years is to make nanocomposites of functional oxides,^16^ where the properties of the individual components can be modified by an appropriate choice of the two phases. Nanocomposites are essentially different from single-phase materials, and offer greater flexibility for obtaining custom-made properties by combining the properties of the two parent phases. Unlike single-phase materials where undesired secondary phases need to be eliminated, the focus while preparing nanocomposites is on obtaining pure bi-phasic compounds. Bi-phasic nanocomposites, where the individual components themselves are complex systems belonging to the family of strongly correlated electron oxide systems, are both interesting as well difficult systems to study. The difficulty lies as much in the synthesis of pure phase nanocomposites as in the understanding of the cross-correlated electronic and magnetic properties. Several synthesis approaches have been tried to create composite oxide nanostructures, often involving techniques that are quite elaborate and costly^17^ as well as requiring high-temperature processing.^18^

With regard to particulate nanocomposites, a relatively easy synthesis route is to physically mix the two phases. However, a simple physical mixing of the two phases leads to clustering and aggregation of the individual phases on the micron scale that is often detrimental to the physical properties of the nanocomposites.^19^ To overcome such issues, different kinds of solution-based chemical approaches have been used yielding nanocomposites with different morphologies such as core–shell nanoparticles and matrix-dispersed composite materials. Samples prepared using chemical approaches often exhibit better homogeneity^20^ than physically mixed samples, as well as better coupling between the constituent phases.^21^ At this stage, developing innovative, scalable, yet low-cost synthesis methods that can yield nanocomposites with the desired physical properties has become crucial.

Sol–gel chemistry is an established method to prepare complex oxide materials, and conventionally, sintering at high temperature is required to obtain phase pure materials. In this work, we report for the first time a unique low-temperature synthesis approach based on sol–gel chemistry for preparing nanocomposites, where we have eliminated the need for further sintering. In this synthesis technique, both the phases of the nanocomposite are formed during the same time. We demonstrate this bottom-up synthesis technique using LaFeO_3_ (LFO) and CoFe_2_O_4_ (CFO) as prototypical magnetic systems. While LFO is a canted G-type antiferromagnet with a high ordering temperature of ∼750 K,^22^ CFO, is a typical ferrimagnet (ordering temperature ∼800 K) exhibiting high saturation magnetization, high coercivity, and large magnetic anisotropy.^23^ The synthesis of LFO, like that of many other complex oxides, requires high-temperature annealing (>500 °C) for phase formation,^24–29^ including the synthesis of nanocomposites where LFO is one of the components.^30,31^ The synthesis technique that we report here is based on the glycine–nitrate combustion synthesis method.^32,33^ For the first time, we use this method that has largely been used to prepare single-phase materials before, to synthesize bi-phasic nanocomposites at a significantly reduced temperature. Remarkably, we observe that each of the two phases (LFO and CFO), instead of hindering the crystallization of the other, facilitate the synthesis process so that phase pure nanocomposites are obtained at temperatures much lower than that required for the crystallization of individual LFO. In the following sections, we will first present the detailed synthesis followed by the structural, morphological, and magnetic characterization of the nanocomposites.

## 2. Synthesis and experimental techniques

### 2.1 Synthesis

In a typical synthesis process of the LFO–CFO nanocomposites, two separate sols were first prepared by dissolving stoichiometric amounts of the precursors of LFO *i.e.*, La(NO_3_)_3_·6H_2_O and Fe(NO_3_)_3_·9H_2_O (Sigma-Aldrich), and the precursors of CFO *i.e.*, Co(NO_3_)_2_·6H_2_O and Fe(NO_3_)_3_·9H_2_O (Sigma-Aldrich), respectively, in distilled water at room temperature. To these solutions, glycine was added to act as the chelating agent (number of moles of glycine = total number of moles of cations). The two clear solutions were then mixed at room temperature and stirred for 20 min. The volume of each solution used to prepare the composite sol was dictated by the desired phase fraction of the composite. For example, to prepare an LFO(50)/CFO(50) nanocomposite, equal volumes of the LFO and CFO sols were mixed in a beaker to form the composite sol. The composite sol was then heated on a hot plate to 80 °C and stirred for 20 min. Next, the temperature of the hot plate was increased and maintained at ∼150 °C till the formation of a gel. Finally, the temperature of the hot plate was increased up to ∼250 °C when a self-combustion reaction with flame occurred, yielding a fluffy powder. No further annealing at higher temperature was performed. Three nanocomposites with phase fractions, LFO : CFO = 95 : 05, 75 : 25, and 50 : 50, were prepared. We refer to these in the rest of the article as LFO(95)/CFO(05), LFO(75)/CFO(25), and LFO(50)/CFO(50), respectively. For comparison, the end members, LFO and CFO, were also prepared using only the LFO and CFO sols, respectively.

### 2.2 Characterization techniques

Differential thermal analysis (DTA) and thermogravimetric analysis (TGA) were performed using a LabsysEvo 1600 DTA/TGA (Setaram). Approximately 1.5 mg and 5 mg of the dried gel (before self-combustion) and sample (after self-combustion), respectively, were placed in an alumina crucible and heated from 30 to 800 °C, at 10 °C min^−1^ under an O_2_ atmosphere (20 ml min^−1^). The DTA and TGA curves were elaborated using the dedicated software Calisto (Setaram).

The samples obtained after self-combustion were characterized by X-ray powder diffraction (XRPD) using a D-5000 diffractometer with CuK_α_ radiation operating at 40 kV and 30 mA. The data were collected in the range 2*θ* = 20–70°, with a step size of 0.02°. Rietveld analysis was performed on the nanocomposites using MAUD.^34^

Transmission electron microscopy (TEM) analysis was performed on the samples obtained after self-combustion using a Philips CM200 microscope operating at 200 kV and equipped with a LaB_6_ filament. For TEM observations, the samples, in the form of powder, were prepared using the following procedure. A small quantity of powder was dispersed in ethanol and subjected to ultrasonic agitation for approximately one minute. A drop of suspension was deposited on a commercial TEM grid covered with a thin carbon film. Finally, the grid was kept in air until complete evaporation of the ethanol.

Fourier-transform infrared spectroscopy (FTIR) spectra were acquired with a Shimadzu IRPrestige-21, equipped with a Specac Golden Gate Single Reflection Diamond Attenuated total reflection (ATR). All samples were analyzed in the region between 4000 cm^−1^ and 450 cm^−1^.

Physisorption with N_2_ at 77 K was performed using an ASAP2020 MP Plus (Micromeritics, USA) equipped with the MicroActive Software for data acquisition and elaboration. The samples were evacuated at 180 °C for 4 h. The specific surface area was evaluated by the well-known Brunauer–Emmett–Teller (BET) method and the pore size distribution (PSD) was calculated using the Barrett–Joyner–Halenda (BJH) method.

Magnetic field-dependent magnetization of the samples was collected using a superconducting quantum interference device (SQUID) magnetometer from Quantum Design Inc. Magnetic hysteresis loops were recorded at *T* = 5 K and 300 K in the ±5 T field range.

## 3. Results and discussion

### 3.1 Investigation of the synthesis process

**Fig. 1:**
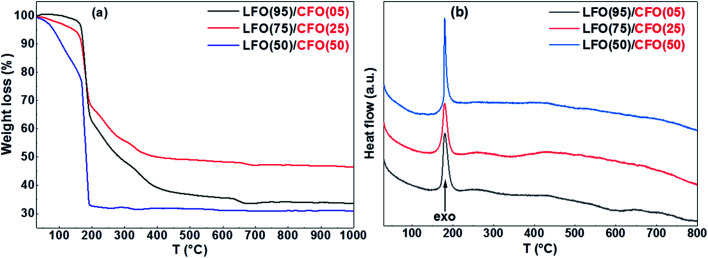
(a) TG and (b) DTA curves of the LFO/CFO gels (before self-combustion).

The TG and DTA curves of the dried gels (before self-combustion) are shown in Fig. 1. The TG curves reveal that for all the gels, irrespective of the fraction of the LFO and CFO phases, the self-combustion process ends at 200 °C, as indicated by a sharp weight loss at ∼200 °C (Fig. 1a). At higher temperature, further variation in the TG curves was observed for the gels of LFO(95)/CFO(05) and LFO(75)/CFO(25) (black and red curves), while for the gel of LFO(50)/CFO(50), no further weight loss was observed (blue curve). The corresponding DTA curves showed a strong exothermic peak centered at 180 °C for all the samples (Fig. 1b). This is the first indication that the reaction occurs below 200 °C, thus offering the possibility of maintaining a low synthesis temperature. The TG curves obtained on the samples after self-combustion are shown in the ESI (Fig. S1†). The TG curves reveal a progressive decrease of the weight loss with an increase in the CFO amount. For the gel of LFO(95)/CFO(05), the weight loss is ∼25% of the initial weight (black curve in Fig. S1†), indicating the presence of a certain amount of intermediary products and amorphous phases that react to form the desired crystalline phases only at higher temperature. The weight loss is significantly reduced for the gel of LFO(75)/CFO(25) (red curve in Fig. S1†), and no variation in the TG curve is observed for the gel of LFO(50)/CFO(50) (blue curve in Fig. S1†).

FTIR spectra recorded on the nanocomposite samples (Fig. S2 in the ESI†) confirm the observations made from thermogravimetric analysis. A fine analysis of the spectra is not easy in these kinds of systems. Nevertheless, the signal in the regions 1200–1600 cm^−1^ and 3100–3600 cm^−1^ can be clearly attributed to the presence of glycine residuals in the samples.^35^ As expected, the intensity of the peaks increases considerably on decreasing the percentage of CFO, thereby confirming that the presence of CFO promotes the formation of the LFO phase. It should be underlined that the presence of a strong signal at ∼2400 cm^−1^ is due to atmospheric CO_2_ due to the AT experimental conditions. The exact composition of the amorphous phases can be probed using detailed elemental analysis. This is, however, out of the scope of the present paper.

### 3.2 Structural and morphological characterization

**Fig. 2:**
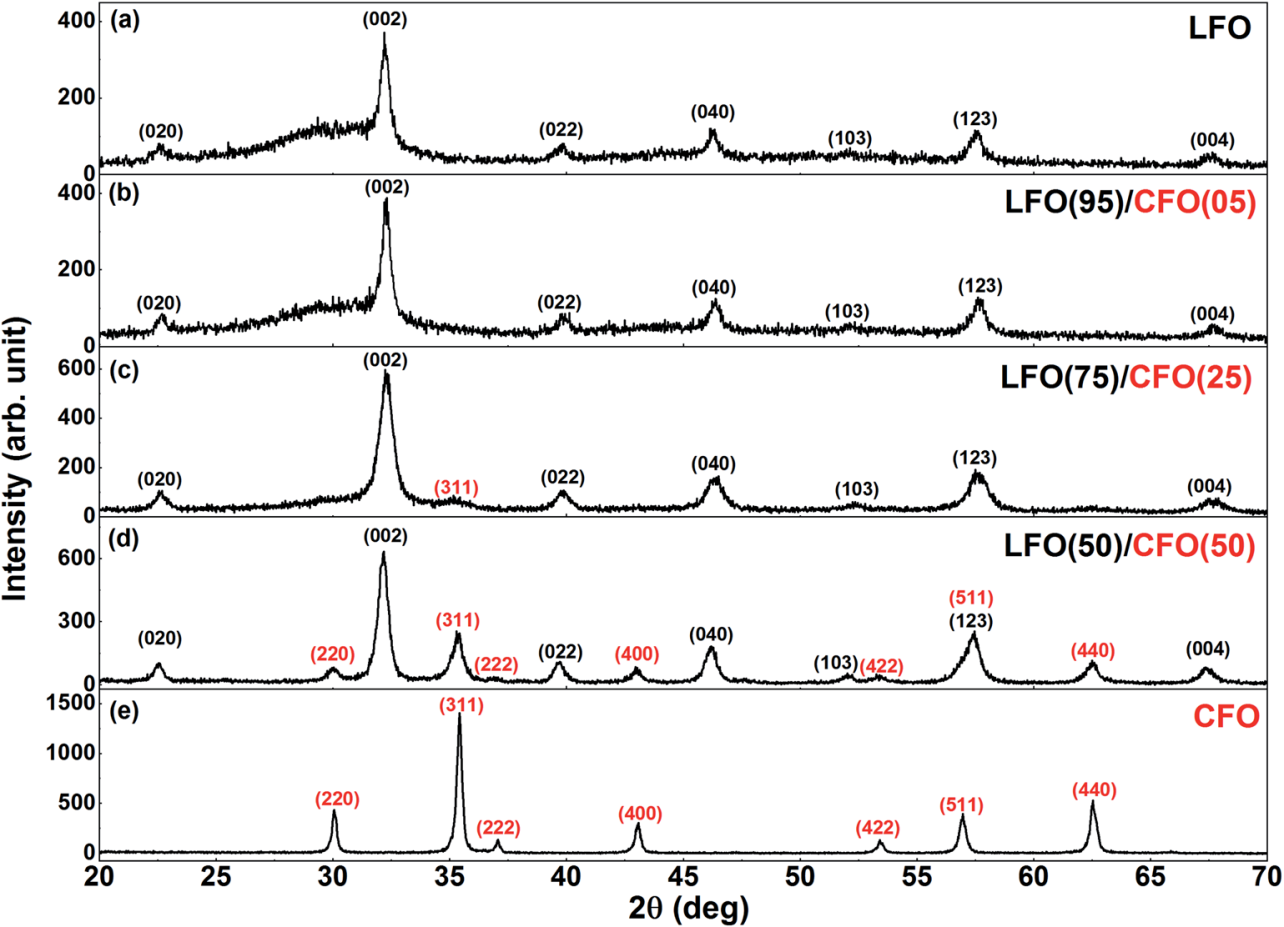
XRPD patterns of (a) LFO, (b) LFO(95)/CFO(05), (c) LFO(75)/CFO(25), (d) LFO(50)/CFO(50), and (e) CFO, after self-combustion. The reflections corresponding to the LFO and CFO phases have been indexed in black and red, respectively.

The XRPD patterns of the as-synthesized samples are shown in Fig. 2. Fig. 2a shows the XRPD pattern of the as-synthesized LFO *i.e.*, the sample obtained after self-combustion. The reflections corresponding to the orthorhombic structure of LFO (s.g. *Pnma*) can be observed and have been indexed in Fig. 2a. However, we also observe a broad shoulder from ∼25–30° that indicates the presence of amorphous material in the sample. This is not unexpected since earlier studies have shown that it is necessary to anneal the as-synthesized sample at ∼500 °C or higher for complete crystallization of LFO^24–29^ and for the complete combustion of organic precursors. In the absence of high-temperature annealing, the XRPD pattern of the LFO sample shows the expected presence of amorphous content. The XRPD pattern of the as-synthesized CFO is shown in Fig. 2e. All reflections could be indexed to the cubic space group (s.g. *Fd*3̄*m*), without the presence of any amorphous content. This is consistent with our earlier studies that have shown that the synthesis of pure phase crystalline CFO does not require high-temperature annealing.^36^ In Fig. 2b, we can see the XRPD pattern of LFO(95)/CFO(05). The pattern looks very similar to that of the end-member LFO. The presence of CFO is not detected because of the small % of CFO in the sample. However, interestingly, the broad shoulder between 25–30° corresponding to the amorphous content in the sample seems to have decreased in the as-synthesized LFO(95)/CFO(05) sample in comparison with that of the end-member LFO. With an increase in the CFO content to 25%, the most intense reflection (311) of the CFO phase is visible in the XRPD pattern (Fig. 2c), and is indexed in red. However, the more interesting point to note is that the broad shoulder between 25–30° corresponding to the amorphous content in the sample shows a clear decrease compared to that in the samples with lower CFO fraction (Fig. 2a and b). We note that characteristic reflections corresponding to CFO do not appear in the XRPD patterns even for LFO(95)/CFO(05) and LFO(75)/CFO(25) samples annealed at higher temperatures, except for the most intense CFO peak in the LFO(75)/CFO(25) samples (Fig. S3 in the ESI†), confirming that their absence is due to the low content of CFO in the samples, and not due to its incomplete crystallization. Fig. 2d shows the XRPD pattern of the LFO/CFO nanocomposite with a further increase in the CFO content (50%). Reflections corresponding to both LFO and CFO are visible (s.g. *Pnma* and *Fd*3̄*m*, respectively) and are indexed in black and red, respectively. Most notably, the broad shoulder between 25–30° corresponding to amorphous content in the sample has disappeared completely. The XRPD data is in agreement with our conclusions from the thermogravimetric analysis, *i.e.*, in general, the amorphous content is seen to decrease with an increase in the CFO fractions, and in particular, for LFO(50)/CFO(50), the nanocomposite obtained after self-combustion is well-crystallized without any amorphous content.

The Rietveld analysis of XRPD data of the nanocomposites is shown in Fig. S4 in the ESI.† Using these fits, we attempted to quantify the relative LFO and CFO content in the LFO(50)/CFO(50) and LFO(75)/CFO(25) samples *i.e.*, the two nanocomposites where reflections corresponding to both phases can be detected. The weight fractions obtained from the Rietveld refinement in the two samples are 49 : 51(1) and 77 : 23(1), respectively, that are in very good agreement with the nominal weight fraction for the two samples. Notably, for the LFO(75)/CFO(25) sample, it was necessary to include an amorphous hump in the background during the Rietveld refinement, while for the LFO(50)/CFO(50) sample, no such amorphous hump was necessary for the refinement. This confirms that for the LFO(50)/CFO(50) sample, all the precursors have crystallized to form the two desired phases (LFO and CFO), while for the LFO(75)/CFO(25) sample, some amorphous material is present. Since reflections corresponding to CFO are not detected in the diffraction pattern of the LFO(95)/CFO(05) sample, it was not possible to quantify the relative LFO and CFO content of this sample using Rietveld refinement.

**Fig. 3:**
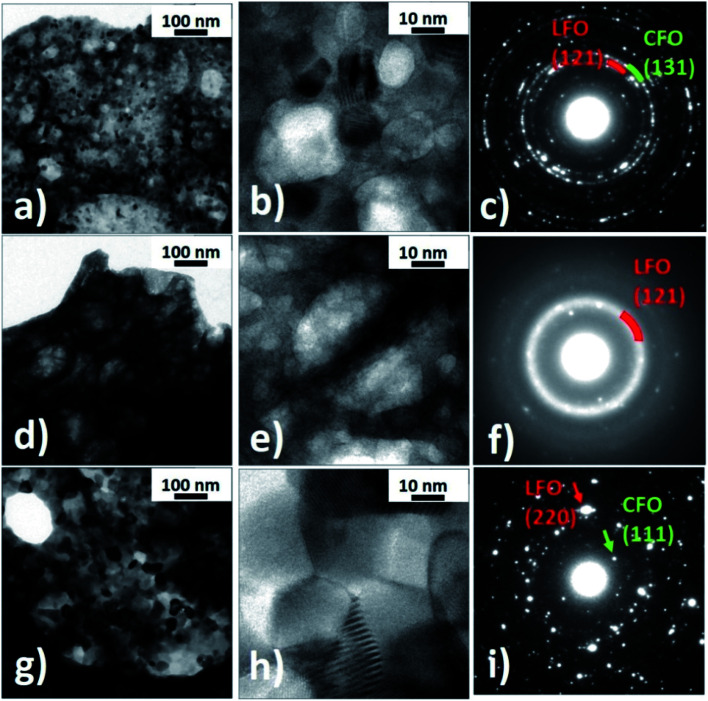
(a–f) LFO(95)/CFO(05) nanocomposite: bright field TEM images (a and b) and (d and e) and corresponding selected area diffraction patterns (c and f); (g–i) LFO(50)/CFO(50) nanocomposite: bright field TEM images (g and h) and corresponding selected area diffraction pattern (i).

Transmission electron microscopy observations were performed to investigate the structure of the nanocomposites in detail. Fig. 3 shows bright field TEM images of the LFO(95)/CFO(05) and LFO(50)/CFO(50) samples and the corresponding diffraction patterns. The LFO(95)/CFO(05) powder has a sponge-like structure with a large number of pores and two kinds of visible regions: one having a large number of nanocrystals (Fig. 3a and b), and the other showing a reduced degree of crystallinity (Fig. 3d and e). In particular, in Fig. 3a, nanocrystals in Bragg condition give rise to a dark contrast (dark spots), while areas corresponding to pores appear brighter. Nanocrystals and pores are better identified in the magnified high-resolution image of Fig. 3b. The regions with a reduced degree of crystallinity are generally more extensive than the former ones and show a very different contrast. Dark spots, corresponding to nanocrystals in Bragg condition, are not visible in Fig. 3d and even at larger magnifications, high-resolution images rarely show the presence of small nanocrystals, Fig. 3e. The different degree of crystallinity is further evidenced by the corresponding selected area electron diffraction (SAED) patterns taken under the same microscope operational conditions. Indeed, Fig. 3c shows well-defined diffraction spots arranged on rings typical of a polycrystalline material, while in Fig. 3f, only few diffraction spots are visible together with some diffraction rings having a diffused intensity typical of an amorphous material. The LFO(50)/CFO(50) powder still has a porous structure, but only areas with a high number of nanocrystals are visible (Fig. 3g and h). The average dimension of these crystals is approximately five times larger than the ones in the previous sample, as can be seen by comparing Fig. 3a, b, g and h. The difference in crystal dimension in the two samples is also reflected in the corresponding SAED patterns. Indeed, Fig. 3i still shows many well-defined diffraction spots as in Fig. 3c, but the selected area being the same for the two samples, a lower number of crystals contributes to the diffraction pattern in Fig. 3i and hence, the diffraction rings are not well defined. Analyzing the interplanar distances associated with the diffraction reflections, it is possible to verify the presence of the LFO and CFO phases in both the samples. In particular, the most intense diffraction contributions of the two phases are shown in Fig. 3c and i. In contrast, in the regions of the sample LFO(95)/CFO(05) showing a lower degree of crystallization, it is possible to observe few diffraction spots attributable to the CFO phase while diffuse rings can be assigned to a poor crystallized LFO phase (Fig. 3f). All these results confirm the conclusions obtained from the XRPD experiments. We have confirmed the presence of pores as seen in the TEM images using nitrogen isotherm analysis at 77 K for all the nanocomposite samples (details in ESI and Fig. S5, S6†). The presence of such pores is a typical characteristic of samples prepared using the self-combustion synthesis technique.

**Fig. 4:**
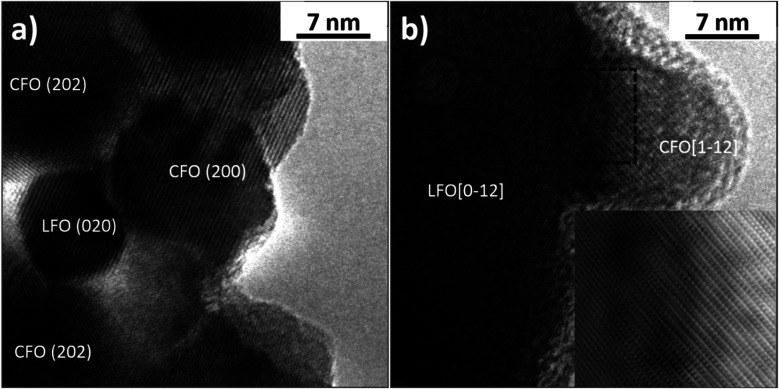
High resolution TEM images showing nanoparticles and atomic planes of (a) LFO(95)/CFO(05) and (b) LFO(50)/CFO(50).

In order to identify the CFO and LFO nanoparticles, high-resolution TEM (HR-TEM) observations were performed on both samples and the images were analyzed by a Fast Fourier Transform (FFT) approach. In particular, Fig. 4a shows some nanoparticles of the two phases and the Miller indexes associated with the visible atomic planes in the LFO(95)/CFO(05) sample. Fig. 4b has been obtained by imaging the LFO(50)/CFO(50) sample, and the crystallographic orientations of the two nanoparticles were identified by the FFT of the image. Generally, it was observed that the CFO nanoparticles have a particle size of approximately 10 nm, much smaller than the particle size of CFO in the absence of an LFO phase (∼70 nm), while the LFO nanoparticles have a size of ∼10 nm and ∼50 nm in the LFO(95)/CFO(05) and LFO(50)/CFO(50) samples, respectively. It is important to note, that the CFO and LFO nanoparticles tend to share some atomic planes, suggesting an oriented growth of one phase over the other. In more detail, the inset of Fig. 4b shows some atomic planes of the LFO phase that deform in order to match the atomic planes of the CFO phase. This inset was obtained by removing the noise from the image indicated by the dotted square using the Gatan Microscopy Suite GMS3 software.^37^ The typical approach consists of obtaining the FFT of the image, the application of a mask to remove the noise, and an inverse FFT to reobtain the image. The atomic periodicity in the two phases is clearly evidenced in this way, and it is possible to observe the nature of the interface.

### 3.3 Magnetization measurements

**Fig. 5:**
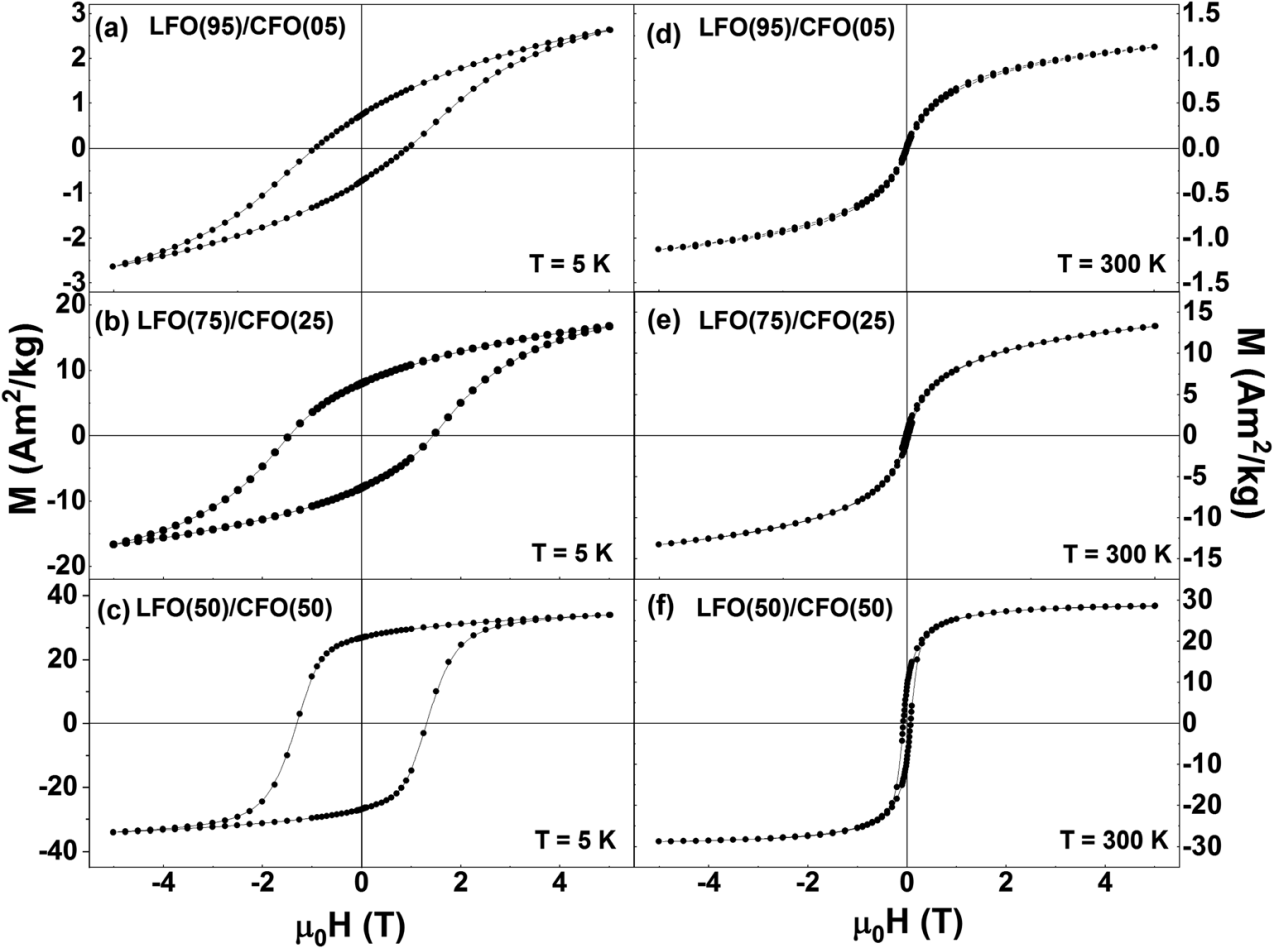
Isothermal magnetization curves of LFO/CFO nanocomposites recorded at (a–c) *T* = 5 K and (d–f) *T* = 300 K.

Fig. 5 shows the isothermal magnetization loops of the nanocomposites recorded at *T* = 5 K (Fig. 4a–c) and *T* = 300 K (Fig. 4d–f). The corresponding magnetization loops of the pure phases (LFO and CFO) are shown in the ESI (Fig. S7†). The single-phase like hysteresis loops indicate that the nanocomposites are strongly exchange-coupled, the shape of the loops significantly changing when the percentage of CFO in the nanocomposites is increased. This change in shape is reflected in a modification of the normalized remanent magnetization *M*_r_/*M*_(5 T)_ (where *M*_r_ = remanent magnetization and *M*_(5 T)_ = magnetization at *μ*_0_H = 5 T) of the samples, which is equal to 0.28, 0.48, and 0.79 for the samples with % of CFO = 5, 25, and 75, respectively (*M*_r_/*M*_(5 T)_ ratios of LFO and CFO = 0.08 and 0.82, respectively). In particular, the *M*_r_/*M*_(5 T)_ value of the 50 : 50 nanocomposite and pure CFO is ∼0.8, as is expected for randomly-oriented particles with cubic anisotropy.

The variation of the magnetic coercivity and magnetization value at *μ*_0_H = 5 T as a function of the percentage of CFO in the nanocomposites are shown in Fig. S8 in the ESI.† The magnetization value at *μ*_0_H = 5 T shows a monotonic increase with the increase in percentage of CFO in the nanocomposites owing to the larger saturation magnetization of pure CFO as compared to that of pure LFO. Interestingly, LFO has been reported to be multiferroic with ferroelectric hysteresis loops^38^ and ferroelastic effect^39,40^ observed at room temperature. By combining antiferromagnetic LFO with a ferrimagnetic spinel system, CFO in this case, which has a reasonably high saturation magnetization, high coercivity, and large magnetic anisotropy,^23^ one can improve the magnetic properties of LFO (as seen here), and thus, expect a promising candidate for applications such as in data storage media, spintronic devices, multiple stage memories, and sensors.^41^

The coercivity values of the nanocomposites exhibit an anomalous trend, with *H*_C_ reaching a maximum for the sample with CFO = 25%, and decreasing for all other samples. Such a maximum in the coercivity of LFO/CFO nanocomposites for a particular composition has been reported before.^30^ This non-monotonic variation of coercivity can be ascribed to the coupling between the antiferromagnetic and ferrimagnetic phases, the strength of which can depend on the relative amounts of the antiferromagnetic and ferrimagnetic phases in the nanocomposites.^42,43^ In addition, the coercivity can also be affected by a change in the magnetic anisotropy due to differences in particle size as well as orientational relationships between the two phases in the nanocomposites.

### 3.4 Mechanism of phase formation and symbiotic effect

The thermogravimetric, structural, and morphological investigations elaborated in the previous sections allow us to develop an insight into the mechanism of phase formation at low temperature in the nanocomposites. We have seen that the as-synthesized LFO(50)/CFO(50) nanocomposite is well-crystallized without any amorphous content despite 50% of the sample consisting of LFO, which normally requires a much higher annealing temperature for crystallization (>500 °C).^24–29^ This is made possible because the formation of CFO proceeds with an exothermic reaction, and the heat produced is sufficient to crystallize the LFO phase at temperatures as low as 200 °C, without the need for further annealing at higher temperatures. HR-TEM observations indicate that the CFO phase acts as a nucleation site for the LFO phase, thereby reducing the energy of formation of the LFO phase. In turn, the LFO phase, growing on the formed CFO nanoparticles, prevents further growth of the CFO nanoparticles in the nanocomposite, as evidenced by the fact that pure CFO (in the absence of an LFO matrix) formed using the same synthesis technique yields particles with a larger size (∼70 nm) as compared to the particle size of CFO in the LFO–CFO nanocomposites (∼10 nm). The effect of the LFO matrix in restricting the growth of CFO particles is further confirmed by estimating the average size of coherent crystalline domains of CFO in the nanocomposites from the Rietveld refinement of the XRPD patterns. Our refinements yield an average crystallite size of 24.8(5) nm and 11.6(9) nm for CFO in the LFO(50)/CFO(50) and LFO(75)/CFO(25) samples, respectively. For the LFO(95)/CFO(05) sample, we were unable to estimate the size of CFO crystals, since we do not see any reflections corresponding to CFO in the XRPD pattern. Notably, the average dimensions of the CFO crystals in the LFO(50)/CFO(50) sample are approximately twice compared to the ones in the LFO(75)/CFO(25) sample. In other words, with an increase in the LFO matrix content, the average dimension of the CFO crystals decreases since the LFO matrix restricts the growth of the CFO particles. To the best of our knowledge, this is the first observation of such a symbiotic phenomenon, where one phase of the nanocomposite helps in the crystallization of the second phase both by providing the heat required for crystallization as well as by reducing the energy of formation of the second phase by acting as nucleation sites, and, in turn, the second phase prevents grain growth of the first phase.

## 4. Conclusions

In summary, we have developed a new composite sol-based synthesis technique for complex oxide nanocomposites, and demonstrated it by synthesizing LFO/CFO bi-magnetic nanocomposites at a significantly reduced synthesis temperature (∼200 °C). The thermogravimetric analysis performed on the dried gels (before self-combustion) confirms that the reaction occurs at 180 °C in all the nanocomposites, irrespective of the relative ratio of the two phases. The XRPD data reveals a well-crystallized as-synthesized LFO(50)/CFO(50) nanocomposite, despite 50% of the sample consisting of LFO, which normally requires a very high annealing temperature for crystallization (>500 °C). This is enabled by our unique synthesis strategy, where we allow both phases of the nanocomposite to form at the same time, thereby utilizing the heat of formation of one phase (CFO) to crystallize the other phase (LFO). Thus, our new synthesis strategy allows us to significantly reduce the temperature (and thereby, the cost) of synthesis by eliminating the need for high-temperature annealing. This is indeed remarkable because most oxides belonging to the family of strongly correlated electron oxides (including pure LFO) require annealing at higher temperatures (>500 °C) for complete crystallization. We also observe a symbiotic effect, where the LFO phase, in turn, restricts grain growth of the CFO phase. Although we have demonstrated our results using prototypical LFO–CFO nanocomposites, it is possible to extend this concept to the synthesis of other bi-phasic nanocomposites as long as the heat of reaction of one phase is sufficient to crystallize the second phase. For example, our studies reveal that replacing CFO with NiFe_2_O_4_ (NFO) leads to very similar results (the XRPD patterns of the as-synthesized LFO–NFO nanocomposites are shown in Fig. S9 in the ESI†). This proves that the synthesis technique reported in this work is not specific to CFO, but rather to the concept of using the heat of reaction generated during the synthesis itself for crystallization and phase formation rather than any external heat source. We believe that this study will provide a new route for scalable, low-temperature synthesis of nanocomposites of different functional complex oxides.

## Conflicts of interest

There are no conflicts of interest to declare.

## Supplementary Material

NA-002-C9NA00619B-s001
