# Supplementary material for: Symbiotic, low-temperature, and scalable synthesis of bi-magnetic complex oxide nanocomposites
Source: Nanoscale Adv. 2020 Jan 16;2(2):851–9. doi: 10.1039/c9na00619b (PMC9417494; doi:10.1039/c9na00619b)
Supplement: NA-002-C9NA00619B-s001 [file NA-002-C9NA00619B-s001.pdf]

Electronic Supplementary Material (ESI) for Nanoscale Advances.
This journal is © The Royal Society of Chemistry 2020

Electronic Supplementary Material for

Symbiotic, low-temperature, and scalable synthesis of bi-magnetic complex oxide nanocomposites

*F. Sayed[1], G. Kotnana[1], G. Muscas[2], F. Locardi[3,4], A. Comite[3], G. Varvaro[5], D. Peddis[3,5], G. Barucca[6], R. Mathieu[1], and T. Sarkar[1]**

[1]Department of Materials Science and Engineering, Uppsala University, Box 534, SE-75121 Uppsala, Sweden

[2]Department of Physics and Astronomy, Uppsala University, Box 516, SE-75120 Uppsala, Sweden

[3]Dipartimento di Chimica e Chimica Industriale, Università degli Studi di Genova, Via Dodecaneso 31, Genova, 16146, Italy

[4]Physics and Chemistry of Nanostructures (PCN), Ghent University, Krijgslaan 281-S3, B9000 Gent, Belgium

[5]Istituto di Struttura della Materia – CNR, Area della Ricerca di Roma1, Monterotondo Scalo, RM, 00015, Italy

[6]Department SIMAU, University Politecnica delle Marche, Via Brecce Bianche, Ancona, 60131, Italy

***Corresponding author:** tapati.sarkar@angstrom.uu.se

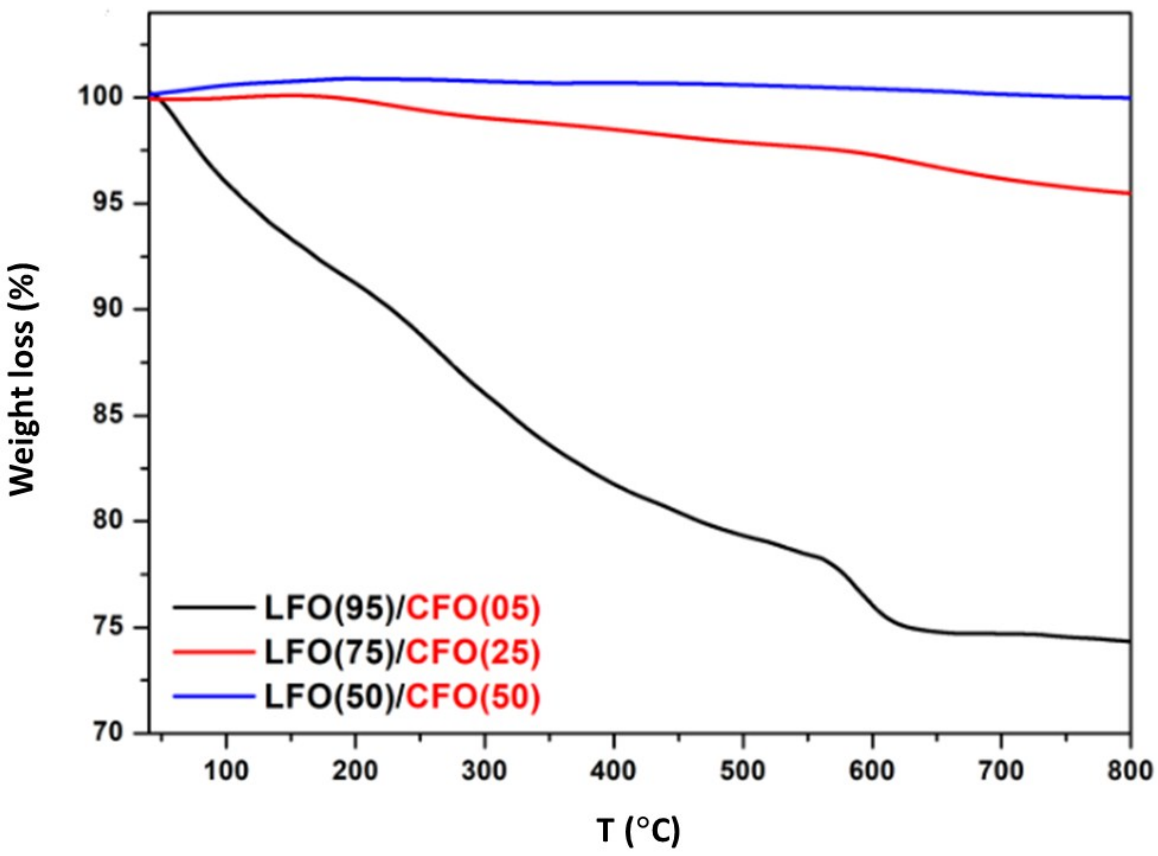

Fig. S1. TG curves of LFO/CFO nanocomposites after self-combustion.

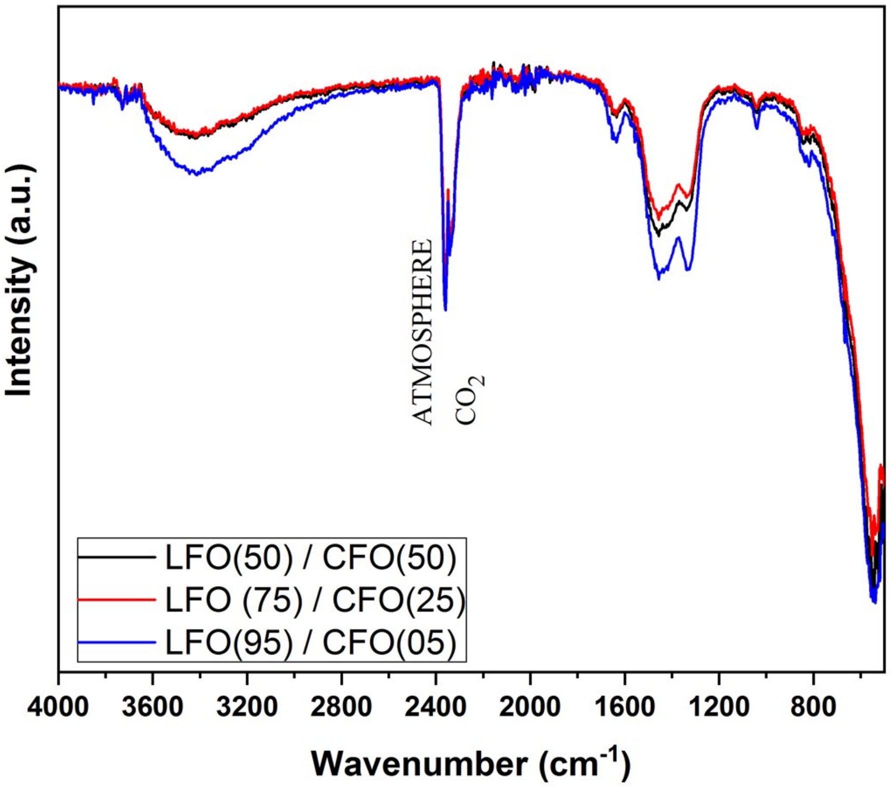

Fig. S2. FTIR spectra of LFO/CFO nanocomposites after self-combustion.

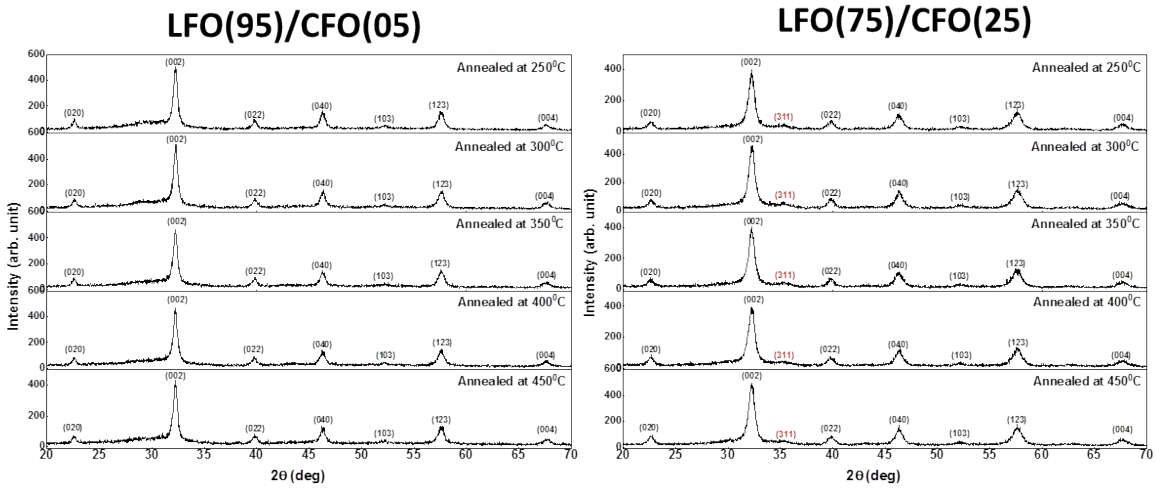

Fig. S3. XRPD patterns of LFO(95)/CFO(05) and LFO(75)/CFO(25) samples annealed at different temperatures ranging from 250–450°C.

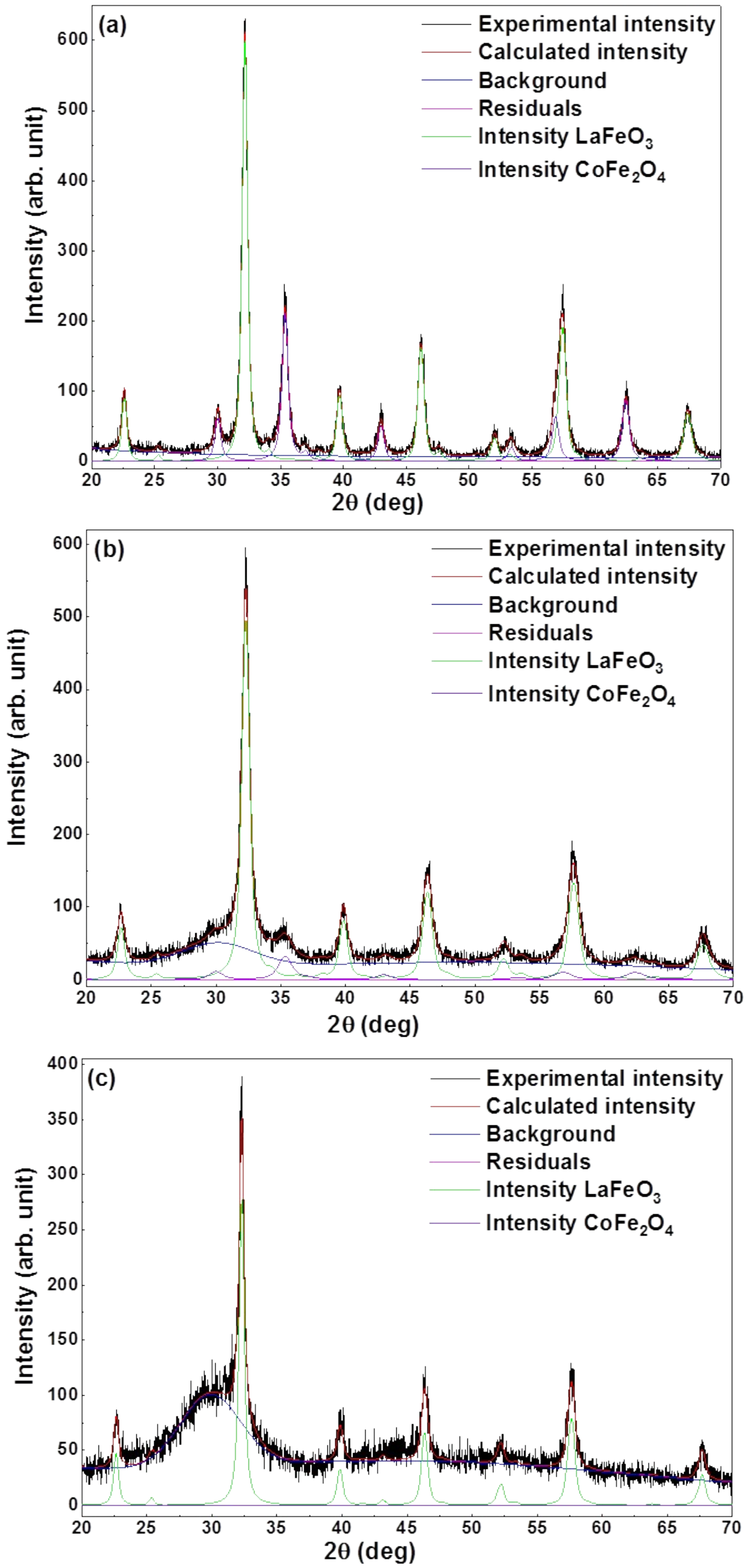

Fig. S4. XRPD patterns of (a) LFO(50)/CFO(50), (b) LFO(75)/CFO(25), and (c) LFO(95)/CFO(05) along with the Rietveld refinement fits and residues.

Fig. S5 shows the N_2 physisorption isotherms of the three nanocomposite samples at 77 K. The adsorption branch of the isotherms is shaped as the type II isotherm of IUPAC classification (Recommendations 1984) typical of macroporous supports. On the other hand, the hysteresis loops indicate the presence of mesopores (pore size between 2 and 50 nm). The type of hysteresis loop is H3 as indicated again by IUPAC. As suggested by Sing et al. [K.S.W. Sing and R.T. Williams, Physisorption Hysteresis Loops and the Characterization of Nanoporous Materials, Adsorption Science & Technology, Vol. 22, No. 10, 2004, 773-782], the isotherm can be classified as the pseudo-type II isotherm instead of type IV since the H3 loops do not show an expected plateau at high relative pressures. The pseudo-type II isotherm of these samples can be associated with their macroporous nature and the hysteresis shape can be related to the metastability of the adsorbed multilayer. By applying the t-plot method, a well-defined mesopore volume could not be estimated. By comparing the three isotherms, the LFO(50)/CFO(50) sample showed the highest adsorbed volume, while the LFO(95)/CFO(05) sample the lowest one. The specific surface area evaluated using the BET method was comparable between the LFO(50)/CFO(50) and LFO(75)/CFO(25) samples having 22.7 m^2/g and 24.6 m^2/g, respectively. The LFO(95)/CFO(05) showed the lowest BET specific surface area (approximately 7.4 m^2/g).

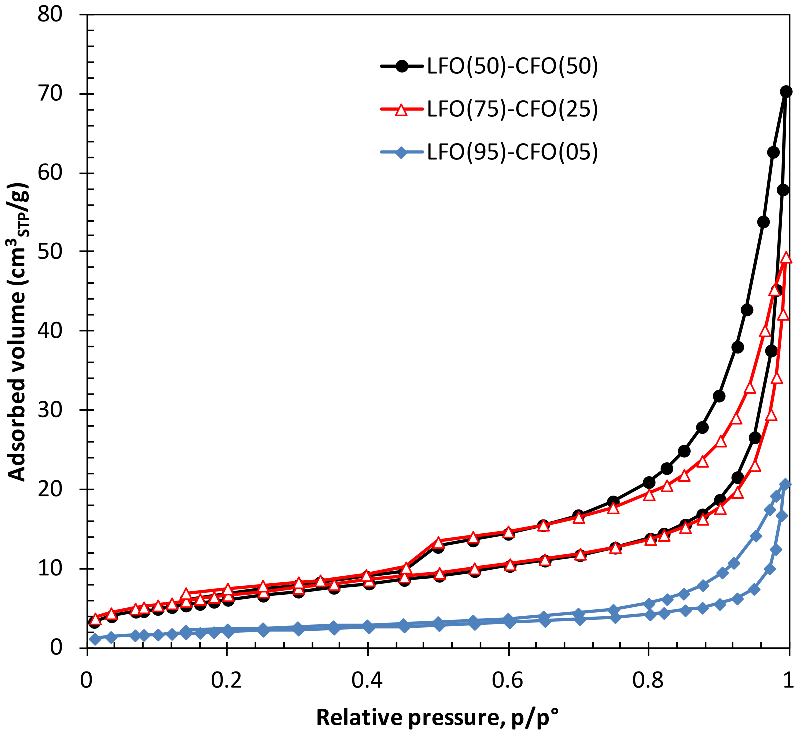

Fig. S5. N_2 isotherms at 77 K of the three nanocomposite samples.

Fig. S6 shows the pore size distribution (PSD) for mesopores calculated using the BJH method applied to the desorption branch of the hysteresis loops. The spike at approximately 3.8 nm should not be considered since it is an artifact due to the step closure of the hysteresis. All the three samples showed a wide PSD in the mesopore range. The PSD of both the LFO(50)/CFO(50) and LFO(95)/CFO(05) samples showed a maximum at approximately 10.5 nm, while the PSD maximum of the LFO(75)/CFO(25) sample was slightly moved to lower pore diameters (approximately 4.5 nm). The physisorption results confirm the presence of pores in the mesopore range observed by transmission electron microscopy.

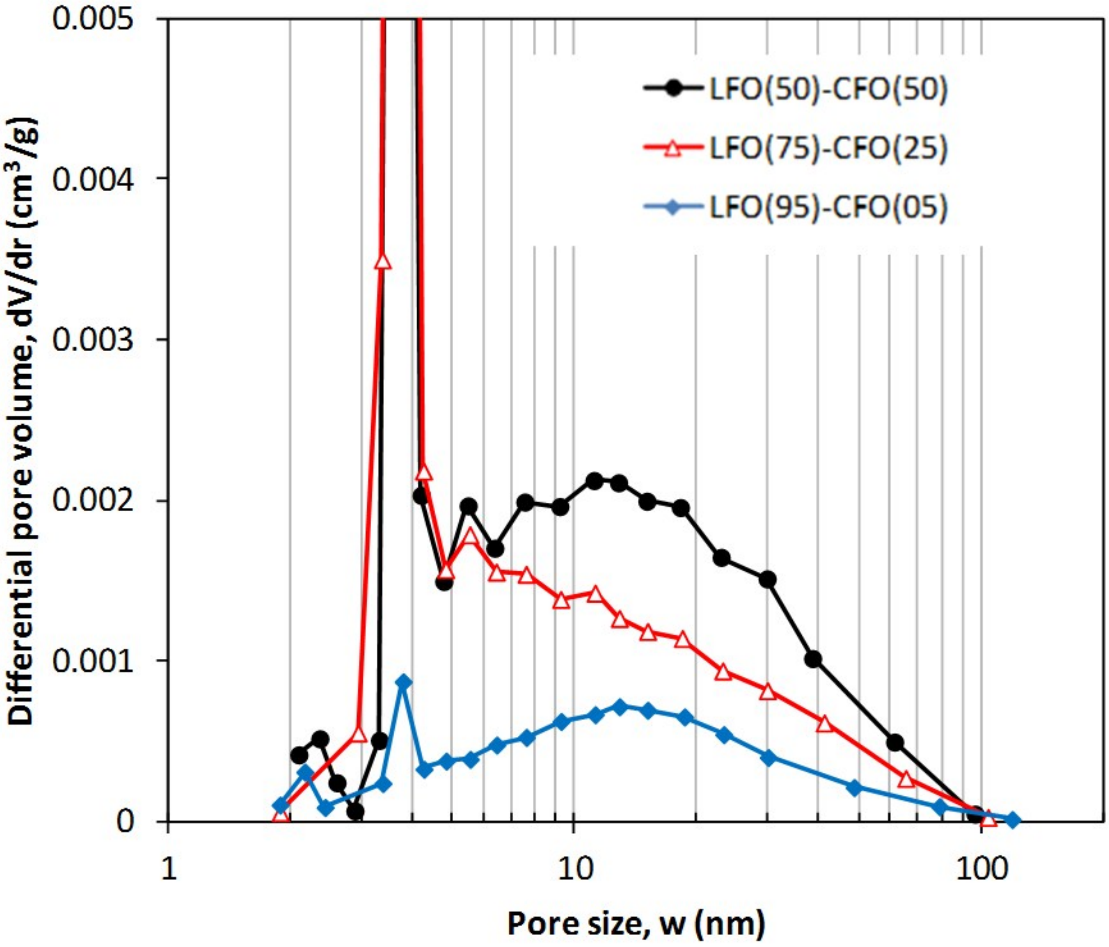

Fig. S6. Pore size distribution obtained using the BJH method applied to the desorption branch of the N_2 isotherms.

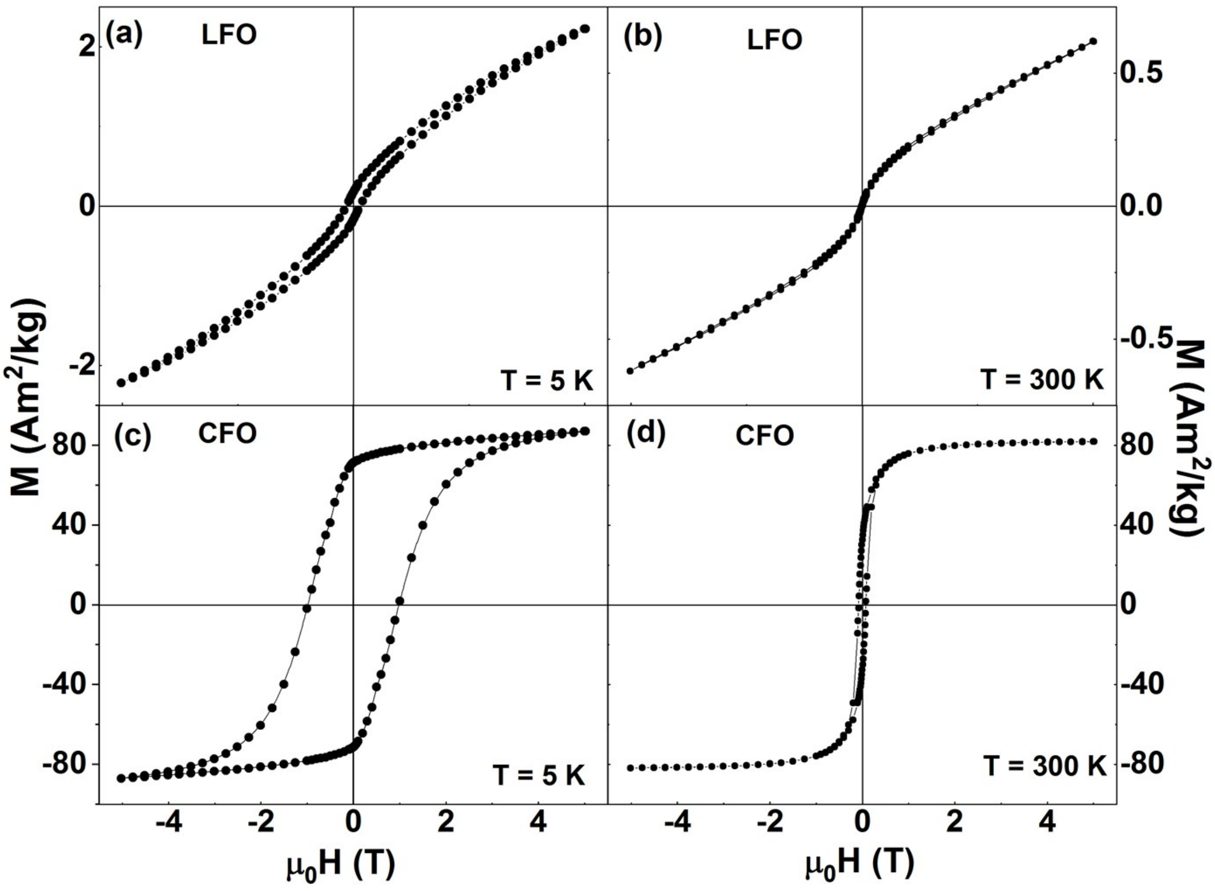

Fig. S7. Isothermal magnetization curves of LFO recorded at (a) T = 5 K and (b) T = 300 K, and CFO recorded at (c) T = 5 K and (d) T = 300 K.

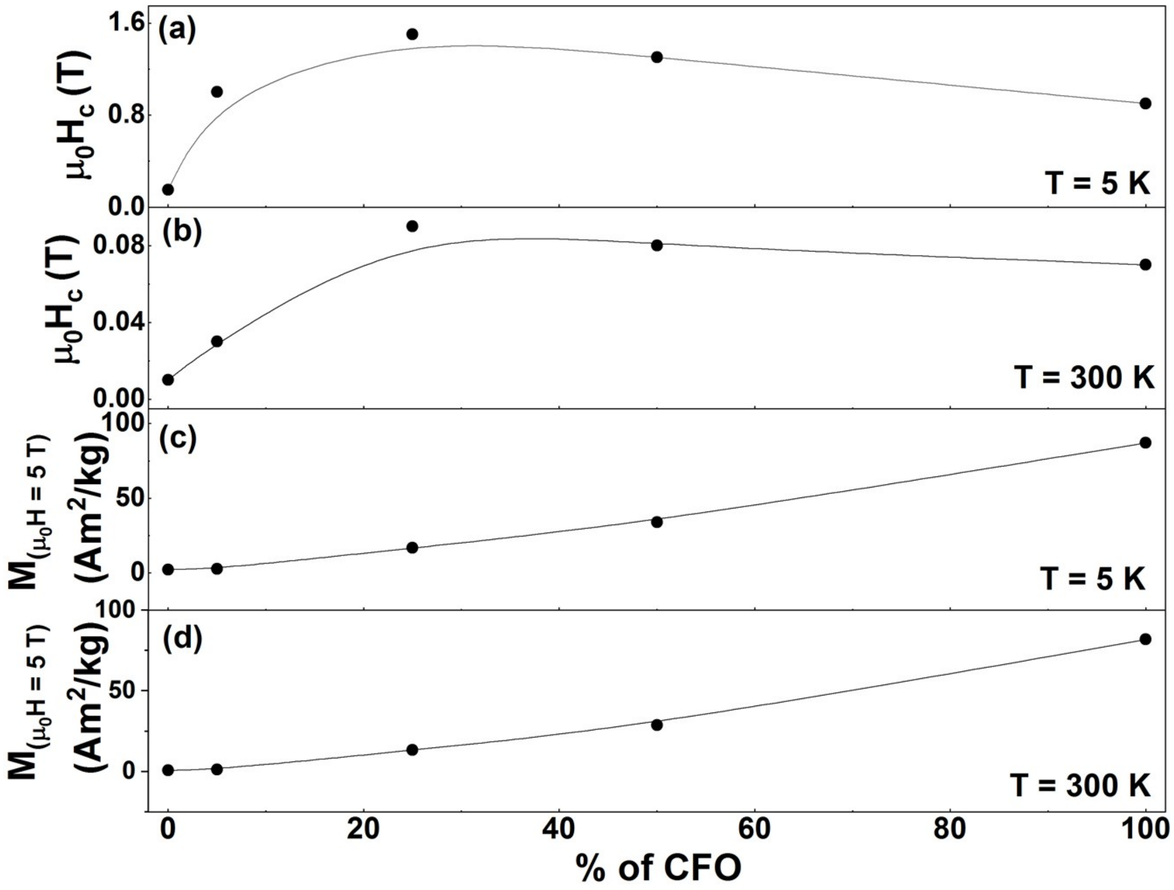

Fig. S8. Variation of (a, b) coercivity and (c, d) magnetization value at μ_0H = 5 T as a function of the % of CFO in the nanocomposites.

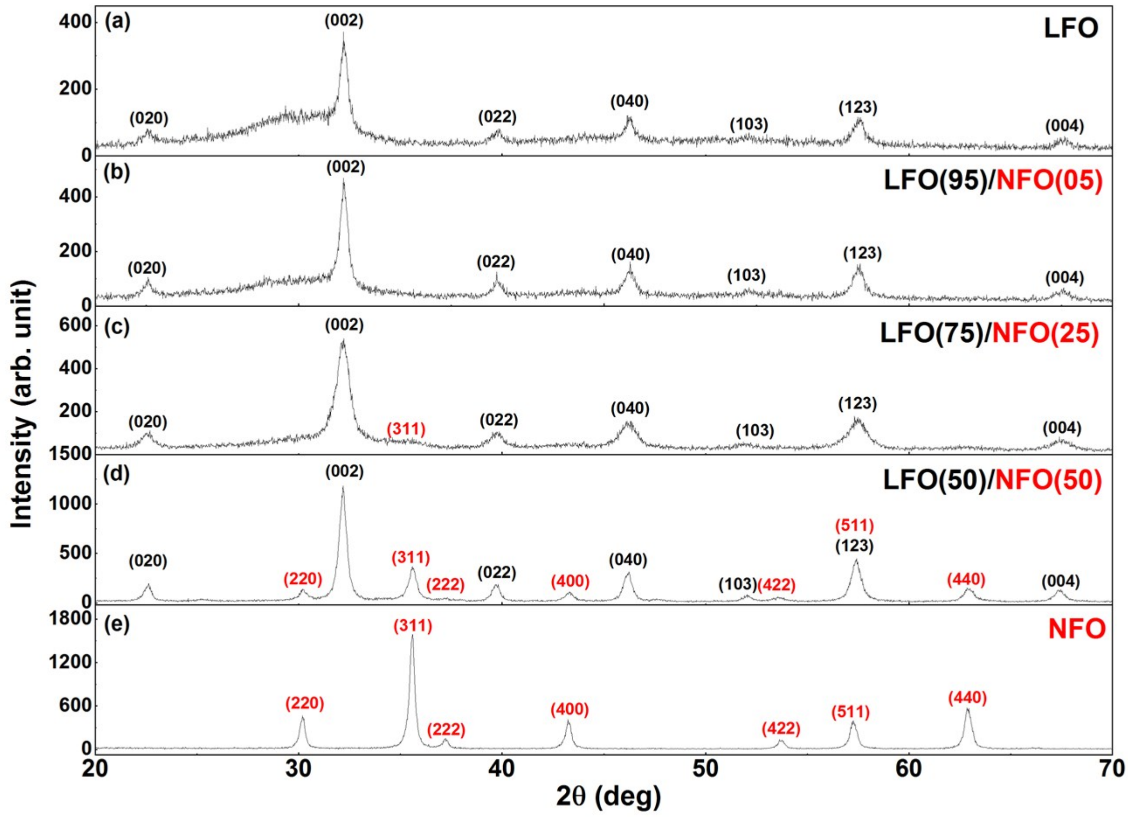

Fig. S9. XRPD patterns of $LaFeO_3$(LFO)/$NiFe_2O_4$(NFO) nanocomposites: (a) LFO, (b) LFO(95)/NFO(05), (c) LFO(75)/NFO(25), (d) LFO(50)/NFO(50), and (e) NFO, after self-combustion. The reflections corresponding to the LFO and NFO phases have been indexed in black and red, respectively.
